# Long Term Fatigue Behavior of Zirconia Based Dental Ceramics

**DOI:** 10.3390/ma3052975

**Published:** 2010-04-28

**Authors:** Moustafa N. Aboushelib

**Affiliations:** Dental Biomaterials Department, Faculty of Dentistry, Champolion Street, Alexandria University, Egypt; E-Mail: info@aboushelib.org; Tel.: +20123000853 Fax: +2035600466

**Keywords:** surface damage, surface roughness, fatigue, flexure strength

## Abstract

This study evaluated the influence of cyclic loading on zirconia bar-shaped specimens after being subjected to three different surface treatments: particle abrasion with either 50 μm or 110 μm alumina and grinding with diamond points, while polished specimens served as a control. Statistical analysis revealed significant reduction (38-67%) in flexure strength (P < 0.001) after three million cycles of dynamic loading for all surface treatments. Scanning electron imaging revealed grain boundary thickening, grain pull-out, and micro-cracking as the main structural defects. The results suggest that various surface treatments of zirconia based dental ceramics may significantly influence their long term fatigue resistance in the oral environment.

## 1. Introduction

In the dental field, there is a trend to shift towards metal free restorations due to concerns about possible biological side effects of metallic alloys. Glass ceramic restorations became an interesting alternative because of their well known biocompatibility and superior esthetics, but the low mechanical properties and brittleness of glass ceramic materials represent the main drawback that has faced their widespread use, especially in high load bearing areas (the posterior region of the mouth). Few years ago, the application of glass ceramic materials was limited to small-sized restorations which are not subjected to high stresses, impact shock, or lateral forces since otherwise the restorations had a high risk of fracture during function [1]. Polycrystalline ceramics made of a fully dense or porous alumina cores which later become infiltrated with glass were used to enhance the mechanical properties of all-ceramic restorations [2,3].

The introduction of zirconia to the dental field opened the design and application limits of all-ceramic restorations. Thanks to its superior flexure strength and high fracture toughness, long span (multi-unit large restorations) and complex zirconia frameworks are now possible with high reliability and clinically proven success rate [4,5,6]. The fabrication of zirconia restorations uses state of the art CAD/CAM systems which require nothing more than few keyboard clicks in order to fabricate complex and accurate restorations [7].

In the biomedical field zirconia is used as hip prosthesis replacing femur heads and as dental implants used to replace missing teeth. There are currently lots of concerns about some drawbacks of zirconia as there are several reports indicating unexpected incidences of fracture of zirconia femur heads [8] which were related to improper handling during the sterilization procedure of these implants [9] and to low temperature phase transformations [10,11]. Accordingly, there are also several studies reporting fracture of zirconia dental restorations after short periods of service. Despite the fact that the flexure strength of zirconia (1,200 MPa) exceeds the average functional stresses expected during function; the estimated failure stress of these broken restorations was much lower compared to the theoretical strength of the material [12,13,14,15].

To understand the failure mechanisms of zirconia based materials, it is necessary to understand both its fabrication technique and its internal structure. Zirconia is a glass free ceramic material that possesses high hardness and mechanical properties in its fully dense state. The material is usually fabricated by different shaping and milling procedures in its partially dense state (green body) that has mechanical properties almost similar to gypsum products allowing easier and much faster fabrication procedures [16]. Afterwards, the shaped body is subjected to a sintering program (heating the material for few hours between 1,300-1,500 °C) to allow the material to reach its full density and strength [17]. As the sintering process is accompanied by volumetric contraction (18-25%) the size of the fabricated green body is consequently increased to account for such shrinkage [18].

At medium temperatures (<1,000 °C), zirconia is composed of a tetragonal crystal structure that transforms into the more stable monoclinic form upon cooling to room temperature. This self-transformation is accompanied by a volumetric expansion leading to micro-cracking of the sintered structures. To prevent this spontaneous transformation, a secondary phase known as the dopant phase is added to the material to stabilize the tetragonal phase at room temperature [19]. Different dopant phases have been utilized for such purpose as magnesia, titanium, calcium, and cerium, while yttrium remains to be the most widely used stabilizer for zirconia used for biomedical applications [20].

Addition of 3% mol yttrium dioxide partially stabilizes the tetragonal phase allowing stress induced tetragonal to monoclinic transformation upon exposure to mechanical stimulation. The accompanying volumetric expansion exerts compressive forces upon the propagating crack tip leading to increase in the fracture toughness of the material, a property known as transformation toughening [4]. Increasing the percentage of the dopant phase above this critical level leads to full stabilization (preservation) of the tetragonal phase and prevents transformation toughening mechanism while decreasing its concentration leads to increase in the transformation activity which could lead to drastic reduction in material properties [9].

On a microscopic level, zirconia is composed of grains which meet each other at grain boundary regions. The size of the grain structure also influences the mechanical properties and the fatigue behavior of the material. Submicroscopic grains (0.3-0.8 µm) are usually used for biomedical applications. Heating zirconia for prolonged periods above 1,000 °C leads to grain growth and to formation to what is known as the cubic form, which can also utterly influence the long term performance of zirconia based materials [21]. The cubic form interferes with homogenous distribution of the stabilizer material in the structure leading to formation of depletion areas lacking necessary concentration of the stabilizer material.

Recent studies reported fracture of zirconia dental restorations at unexpectedly short service periods and under low loading forces. This failure was related to the amount of surface damage introduced during different laboratory and clinical procedures. Increasing the surface roughness of zirconia frameworks using particle abrasion is a common routine used to enhance bonding of resin adhesives to zirconia [5,22]. A straightforward correlation was established between the type and extent of surface damage and reduction in flexure strength. Moreover, it was found that increase in surface damage in the form of scratches, grooves, and grain pull-out was directly related to reduction in the expected service time of the restoration (calculated using Weibull modulus analysis) [23]. On the contrary, there are studies that did not detect a direct relation between surface damage and reduction in flexure strength, which could be related to the absence of long term cyclic loading under water [24,25,26,27].

At the moment there are no clear guidelines about the ideal surface treatment method of zirconia restorations and the associated influence on their mechanical properties. While some manufacturers recommend rough particle abrasion protocols (large aluminum oxide particles using high pressure) other advice against such procedures. Additionally, the influence of cyclic loading and chemically assisted crack propagation on the long term performance of zirconia restorations and their interaction with surface damage remain unclear. These parameters were addressed in the present study.

## 2. Results and Discussion

Statistical analysis revealed significant differences in the flexure strength of the tested specimens before and after cyclic loading (F = 31, *P* < 0.001). Subjecting the specimens to dynamic fatigue under water resulted in significant reduction in flexure strength with polished specimens demonstrating the least reduction (38%). Particle abrasion with coarse alumina particles at high pressure and grinding with diamond points resulted in the highest reduction in flexure strength (67%). Previous data are summarized in Table 1.

**Figure 1:**
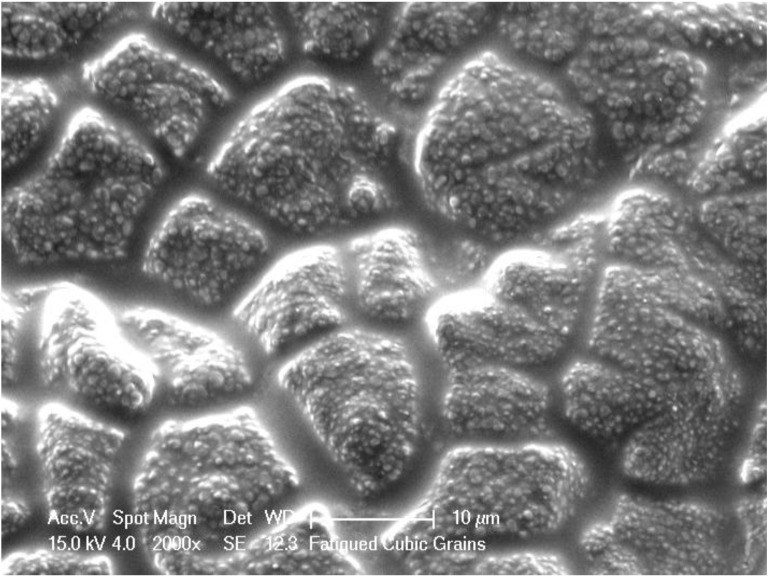
SEM image, 2,000×, demonstrating thickening of grain boundaries after cyclic loading observed for all examined specimens.

**Figure 2:**
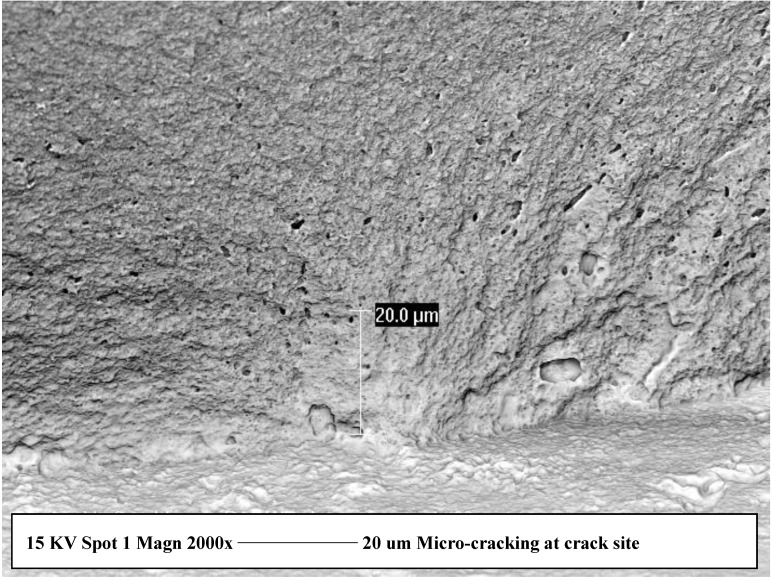
Secondary electron imaging, 1,000×, back scatter mode revealing micro-cracking appearing as black areas in the structure at location of failure site.

SEM examination of the fracture surface indicated that the critical crack (origin site of the fracture) was located at the tensile surface. Airborne particle abrasion resulted in creation of sharp scratches and grooves, while grinding produced parallel lines on the ground surface. Cyclic loading resulted in grain boundary thickening (Figure 1) and micro-cracking (Figure 2) in the region of the critical crack. Four point bending is one of the most commonly used methods to evaluate the flexure strength of the material under evaluation. Compared to other flexure tests such as three-point bending or biaxial flexure, it has the advantage of testing larger surface area of the specimen which is not influenced by the stress concentration problem around the supporting rollers. In combination with cyclic loading under water the test can help to accurately predict the long term performance of the tested material [29]. Compared to other studies [30,31,32,33] a significantly higher number of dynamic cycles were used (3 million) but at relatively lower stress range (20% of the theoretical strength). The rationale behind such a fatigue test was to prevent generation of a crack tip due to high stress concentration which would gradually propagate leading ultimately to fracture of the specimens after a low number of cycles. On the other hand, cyclic loading at low stresses has a better capacity to detect structural weakness of the material and to explore the long term failure mechanism under function.

**Table 1:** Percent of reduction in flexure strength after cyclic loading compared to theoretical strength of polished group. Groups with similar capital superscript letters indicate no significant statistical difference in flexure strength at same test interval.

Test group	Initial flexure strength (MPa)	Flexure strength after cyclic loading (MPa)	Percentage of strength reduction in relation to polished specimens (1208 MPa)
Particle abrasion (50 µm)	1131 ± 131 ^A^	655 ± 155 ^C^	45%
Particle abrasion (110 µm)	720 ± 187 ^B^	388 ± 193 ^D^	67%
Grinding	769 ± 166 ^B^	409 ± 188 ^D^	66%
Polishing	1208 ± 97 ^A^	748 ± 88 ^C^	38%

Secondary electron imaging revealed thickening of the grain boundary region which suffered from micro-cracking and pull-out of some surface grains. The grain boundary regions of zirconia based materials play an important role affecting slow crack growth, low temperature aging, and diffusion of different dopant materials [10,34,35]. This form of structural damage would allow easier inter-grain propagation of surface cracks facilitated by the presence of water at a crack tip [36]. Increasing the amount and type of surface damage induced by different surface treatments would increase the number of already available surface cracks ready to propagate at the weakened grain boundary regions. It has also to be mentioned here that micro-cracking will result in gradual accommodation of structural damage (in the form of plastic deformation and deviation in R curve behavior) which will degrade material properties during service.

An interesting observation was that neither of the tested specimens failed during cyclic loading even with the observed grain boundary thickening and micro-cracking which reflects the capacity of the used zirconia in resisting crack propagation and in accommodating structural damage. The reported reduction in flexure strength of zirconia subjected to cyclic loading was similarly observed in previous studies [28,31,33,37], nevertheless there are reports that did not detect any deterioration effect after cyclic loading of zirconia restorations which could be related primarily to the low number of cycles used in these studies [38,39]. Additionally, testing anatomically shaped specimens in the form of crown and bridges will allow other factors to interfere with the applied load as the tooth structure and type of adhesive cement used [39].

The data presented in this study indicate that there is a strong correlation between surface damage and fatigue resistance of the material. Highly polished specimens demonstrated the least reduction in flexure strength after cyclic loading, which could be considered as the theoretical long term performance of the material. Where suitable, a 30% safety factor could be used to predict deterioration in mechanical properties of polished zirconia subjected to cyclic loading. On the contrary, a 60% safety factor could be used when the material is subjected to surface alteration during different phases of the clinical and laboratory procedures. Such precautions will be most relative in thin sections of any zirconia structure subjected to dynamic loading [23]. In a previous study, cyclic fatigue which induced contact surface damage was reported to induce micro-cracking, plastic deformation, and monoclinic transformation of zirconia base materials [40]. Plastic deformation could also lead to grain boundary thickening and alteration of the shape of surface grains.

Previous studies reported that particle abrasion could lead to the formation of deep surface cracks that could extend up to 50 μm from the surface. Such deep cracks would be favorable sites for the initiation of critical cracks especially in the presence of water at the crack tip. The effect of surface damage on the mechanical properties of zirconia was recently evaluated by Wang *et al*., who reported significant reduction in initial flexure strength for surface damaged zirconia restorations. Survival statistics reported that inducing surface damage could lead to shifting of the theoretical failure stress (60% of the stress of polished specimens) to values as low as 40% of the theoretical strength of surface damaged material which could simply explain the reported fractures after short service time [23,41].

In the biomedical field, zirconia implants and prosthetic devices receive different surface treatments and sometimes in combination with different surface coatings to enhance osseo-integration with bone cells. These methods could induce different types of surface damage which could explain unexpected fractures as previously reported [37,42]. Thus the combination of surface damage and cyclic loading under wet conditions seems to be the primary cause of micro-cracking and grain boundary thickening both acting as main failure mechanisms of the tested zirconia restorations [43,44].

## 3. Experimental Section

### 3.1. Preparation of the specimens

Eighty bar-shaped zirconia specimens (2 × 3 × 25 mm) were prepared by cutting partially sintered zirconia blocks (Procera zirconia; NobelBiocare, Göteborg, Sweden) using a precision cutting instrument (Isomet 1000; Buehler, Lake Bluff, IL, USA) and a diamond coated disc (Diamond Wafering Blade, No 11-4254; Buehler). The bars were gently polished on 800 and 1,200 grit silicon carbide paper using a custom made rotating metallographic polishing device. The specimens were finally sintered according to manufacturer’s instructions (6 hour holding time at 1,350 °C).

The bars were divided into four groups which received one of the following surface treatments which represent different surface roughening methods produced by different clinical and laboratory procedures (n = 20):

1) Particle abrasion with 110 μm aluminum oxide particle at 0.3 MPa pressure and 10 mm distance (P-G 400; Harnisch & Rieth, Winterbach, Germany).
2) Particle abrasion with 50 μm aluminum oxide particle at 0.15 MPa pressure and 10 mm distance (P-G 400).
3) Grinding with diamond coated points.
4) Polishing using 0.5 μm diamond paste. This group was used a control to measure the theoretical strength of the used zirconia.

### 3.2. Cyclic loading program

All specimens were subjected to three million dynamic loading cycles in a four point flexure test setup (20 × 10 mm span) under water using a custom made pneumatic loading device. The applied load shifted between a maximum and a minimum load which resulted in alternating flexure stress at the tensile surface of the specimens between 50-200 MPa. The stress at the tensile surface was calculated using 4-point flexure strength using the equation mentioned further in the text.

The selected stress range represent less than 20% of the theoretical flexure strength of the zirconia used in this study. Such low stress cyclic loading program was selected to study long term fatigue behavior and slow crack growth under water [28].

A 0.5 mm thick silicon sheet was placed between the loading point and the surface of the specimens to prevent generation of contact surface damage which could lead to premature failure. The sheet was replaced every 250,000 cycles. The test was conducted under a water bath at 37 °C at rate of 1 cycle/3 sec.

### 3.3. Flexure strength test

Before and after completion of the three million cycles, the specimens were subjected to one cycle load to failure program performed in the same four point flexure test setup in a universal testing machine (Instron 6022, Instron Corp, high Wycomb, England) at 0.5 mm/min crosshead speed. The peak load was extracted from computer generated files and the flexure stress at the tensile surface of the bars was calculated using the following formula:

$$F_{S}=\frac{3F(L-l)}{2wh^{2}}$$

where L and l are the outer and inner spans, respectively, w and h are the specimen width and thickness, respectively (all in meters).

The fracture surface was examined using scanning electron imaging (XL 20; Philips, Eindhoven, The Netherlands) at different magnifications. Secondary electron imaging was conducted by preparing the fracture surface for high magnification imaging. Specimens were ultrasonically cleaned and gold sputter coated (S150B sputter coater; Edwards, Crawley, UK). Electro-conductive material was placed on the carrying stub to prevent electro-charging during examination. The spot size (2 μm), kilo voltage (Kv 15), and distance from electron length (20 mm) were adjusted to provide clear image of the examination field under complete vacuum.

### 3.4. Statistical analysis

Two way analysis of variance (ANOVA) and Bonferroni *post hoc* tests (SPSS 14.0; SPSS, Inc, Chicago, IL, USA) were used for statistical analysis (n = 20, α = 0.05).

## 4. Conclusions

Within the imitations of this *in vitro* study, it can be concluded that cyclic loading in water combined with different surface treatments of zirconia based dental ceramics significantly reduced their mechanical properties and therefore may affect their long term performance.
